# Supplementary material for: Conditional chemoconnectomics (cCCTomics) as a strategy for efficient and conditional targeting of chemical transmission
Source: eLife. 2024 Apr 30;12:RP91927. doi: 10.7554/eLife.91927 (PMC11060718; doi:10.7554/eLife.91927)
Supplement: Figure 3—source data 3. [file elife-91927-fig3-data3.zip › Figure 3 source data3 part 2.zip!/Figure 3-Source data 3-part2/1062-13-CRISPResso_Report_jt28fhr9.zip!/CRISPResso_on_1062_15/2b.gcaggaaatgagctacctac.Nucleotide_percentage_quilt_around_sgRNA_gcaggaaatgagctacctac.pdf]

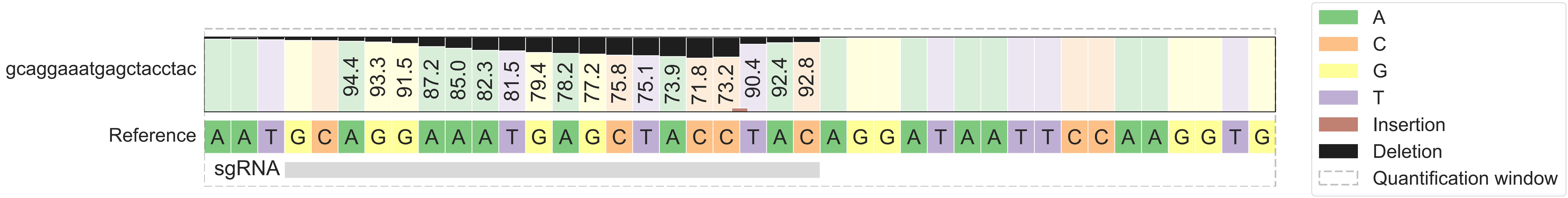

gcaggaaatgagctacctac
94.4
93.3
91.5
87.2
85.0
82.3
81.5
79.4
78.2
77.2
75.8
75.1
73.9
71.8
73.2
90.4
92.4
92.8
Reference
A A T G C A G G A A A T G A G C T A C C T A C A G G A T A A T T C C A A G G T G
sgRNA
A
C
G
T
Insertion
Deletion
Quantification window
